# Deletion Study of DNA Topoisomerase IB from *Leishmania donovani*: Searching for a Minimal Functional Heterodimer

**DOI:** 10.1371/journal.pone.0001177

**Published:** 2007-11-14

**Authors:** Rosario Díaz González, Yolanda Pérez Pertejo, David Ordóñez, Rafael Balaña-Fouce, Rosa M. Reguera

**Affiliations:** Departamento de Farmacología y Toxicología (INTOXCAL), Universidad de León, León, Spain; Swiss Tropical Institute, Switzerland

## Abstract

The substantial differences between trypanosomal and leishmanial DNA topoisomerase IB concerning to their homologues in mammals have provided a new lead in the study of the structural determinants that can be effectively targeted. *Leishmania donovani*, the causative agent of visceral leishmaniasis, contains an unusual heterodimeric DNA topoisomerase IB. The catalytically active enzyme consists of a large subunit (LdTopIL), which contains the non-conserved N-terminal end and the phylogenetically conserved “core” domain, and of a small subunit (LdTopIS) which harbors the C-terminal region with the characteristic tyrosine residue in the active site. Heterologous co-expression of *LdTopIL* and *LdTopIS* genes in a topoisomerase I deficient yeast strain, reconstitutes a fully functional enzyme LdTopIL/S which can be used for structural studies. An approach by combinatorial cloning of deleted genes encoding for truncated versions of both subunits was used in order to find out structural insights involved in enzyme activity or protein-protein interaction. The role played by the non-conserved N-terminal extension of LdTopIL in both relaxation activity and CPT sensitivity has been examined co-expressing the full-length LdTopIS and a fully active LdTopIΔS deletion with several deletions of LdTopIL lacking growing sequences of the N-terminal end. The sequential deletion study shows that the first 26 amino acids placed at the N-terminal end and a variable region comprised between Ala548 to end of the C-terminal extension of LdTopIL were enzymatically dispensable. Altogether this combinatorial approach provides important structural insights of the regions involved in relaxation activity and for understanding the atypical structure of this heterodimeric enzyme.

## Introduction

*Leishmania donovani* is the etiological agent of visceral leishmaniasis, a very serious disease transmitted by the bite of female sandflies of the *Phlebotomine* and *Lutzomyia* genera, characterized by fever, swelling of the spleen and liver and anaemia which may be fatal if not diagnosed and treated on time [1]. It is well-established the role played by the host immune system in resistance and healing of this disease but no effective vaccine has been developed at present. Current pharmacopoeia against leishmaniasis includes amongst others; pentavalent antimonium salts, macrolides, aromatic diamidines and recently alquilphospholipids. Most of them are plenty of undesirable side effects, or require parental administration and long-term treatments [2].

DNA topoisomerases are striking candidate targets for leishmaniasis therapy [3]. These enzymes catalyze changes in the topological state of duplex DNA during replication, transcription, recombination and DNA repair processes, by introducing transient single strain breaks in the nucleic acid backbone. Trypanosomatid type IB DNA topoisomerases differ from their homologues in their oligomeric nature. These enzymes are AB heterodimers with the genes encoding each protomer located on different chromosomes [4]–[5]. Genetic analyses identified a gene for a large subunit, namely *LdTopIL*, on *L. donovani* chromosome 34, encoding for a 636-amino acid polypeptide with an estimated molecular mass of 73 kDa. This subunit contains a sequence resembling the core domain of human topoisomerase I (hTopI from now) and conserves the catalytic “tetrad”: Arg-314, Lys-352, Arg-410 and His-453. *LdTopIS*, the gene encoding for the small subunit, is found on *L. donovani* chromosome 4 and encodes for a 262-amino acid polypeptide with a predicted molecular mass of 28-kDa. The small subunit includes the “SKXXY” signature placed at the C-terminal domain of all type I DNA topoisomerases, which conserves a tyrosine residue playing role in DNA cleavage. Gene silencing studies carried out with the *Trypanosoma brucei* bi-subunit topoisomerase IB reveals that both subunits are required for parasite survival [6].

The structural differences between human and kinetoplastid type IB DNA-topoisomerases make this enzyme an attractive target for chemotherapeutic intervention [7]–[9]. Topoisomerase inhibitors fall into two general categories: compounds that stimulate the formation of covalent enzyme-DNA complexes or topoisomerase poisons (class I inhibitors), and products that interfere with the enzymatic functions of the enzyme (class II inhibitors). Camptothecin (CPT) is a good example of a class I topoisomerase poison. This compound has a strong anti-leishmanial effect in experimental trials [10] and some analogues are used for the treatment of cancer (see the recent review by Pommier [11]). CPT is an uncompetitive inhibitor which, by binding covalently to DNA, traps the enzyme in an immobile ternary complex, preventing the DNA religation step. CPT generates covalent DNA-topoisomerase complexes with both nuclear and kinetoplastic preparations of DNA from trypanosomes, leishmanias [12] and other protozoan parasites of medical importance [13].

Previous studies have shown that the proteolytic cleavage of core and catalytic domains in hTopI within the non conserved linker domain did not affect markedly catalysis. Stewart and co-workers [14] reconstituted the relaxation activity of human topoisomerase by adding to the core domain a series of peptides containing the C-terminal domain, in a proportion of 1∶1 in the presence of DNA. This finding was reinforced by Park and Sternglanz [15] using a two-hybrid expression system. The authors identified proteins containing part of the linker and the C-terminal domain that supplemented the catalytic core of *Saccharomyces cerevisiae* topoisomerase I (yTopI).

The objective of this study is to find out structural insights within the C-extension end of the large subunit needed for a functional interaction with the small subunit. Our findings reveal that 75 residues placed at the C-terminal end of LdTopIL and 169 residues placed at the N-terminal extension of LdTopIS are dispensable for topoisomerization of supercoiled DNA.

## Materials and Methods

### Reagents and culture media

DNA modification and restriction enzymes were provided from Roche (Basel, Switzerland) and Amersham Biosciences. *Pyrococcus furiosus* (*Pfu*) polymerase was from Stratagene (La Jolla, CA, USA). Cell culture media, CPT and other chemicals and reagents were purchased from Sigma (St. Louis, MO). Primers for PCR amplification were from Sigma Genosys (UK).

### Yeast and leishmanial strains

*S. cerevisiae* strains for protein expression: EKY3 [MAT α ura3-52 his3Δ200 leu2Δ1 trp1Δ63 TopIΔ::TRP1] and MBY3 [MAT α ura3-52 his3Δ200 leu2Δ1 trp1Δ63, TopIΔ::TRP1 rad52 Δ::LEU2], both lacking topoisomerase activity, were generously gifted by Dr. MA Bjornsti (St. Jude Children's Research Hospital, Memphis, TN) [16]. *Leishmania donovani* promastigotes (LEM75, Ethiopian) were kindly supplied by Dr. J.M. Requena (Centro de Biología Molecular “Severo Ochoa”, CSIC Madrid, Spain).

### Cloning of leishmanial DNA topoisomerase I

Cloning of heterodimeric *LdTopIL/S* was performed as described previously [4]. *LdTopIL* (GenBank #AF303557) gene, was obtained by the screening of a λ-EMBL3 genomic library [17] whereas gene encoding the small subunit, *LdTopIS* (GenBank # AY062908) was amplified using primers based on *L. major* Friedlin genome Project and *L. donovani* genomic DNA as template [18].

### C- and N-terminal truncations of LdTopIL/S

**Figure 1:**
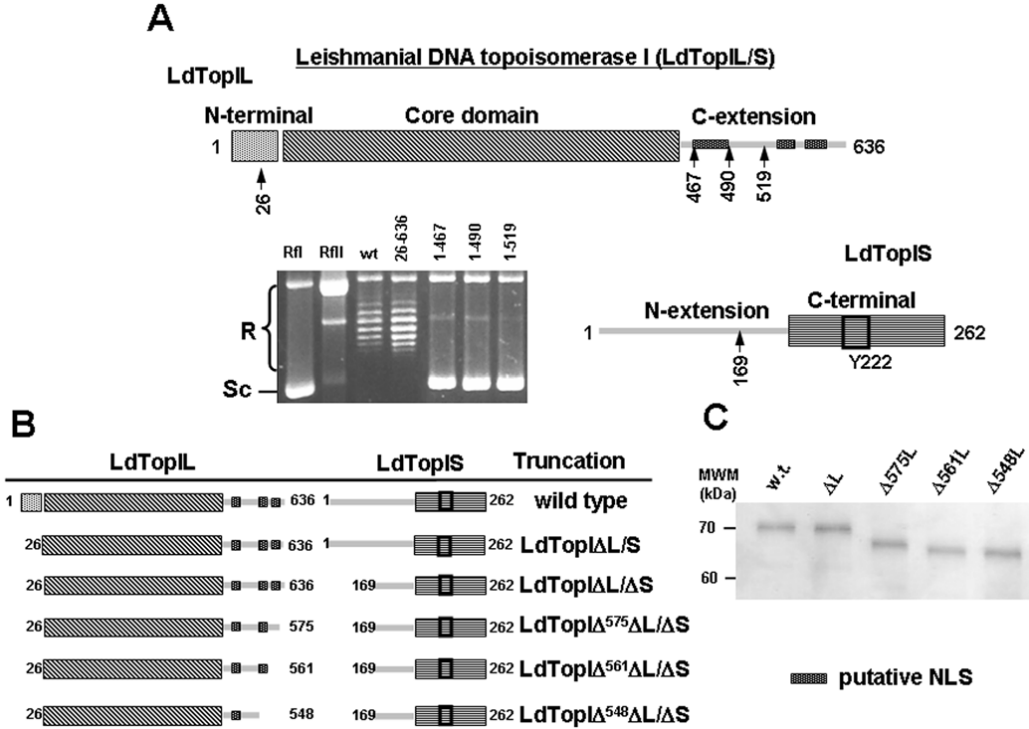
Heterologous expression of LdTopIL/S and deletions in a topoisomerase-deficient yeast EKY-3 strain. A) Deletions performed on gene encoding for LdTopIL were *LdTopIΔL* at the N-terminal end, and *LdTopIΔ^467^L*, *LdTopI^490^L*, and *LdTopI^519^L* at the C-terminal end. These four deletions were cloned together with *LdTopIS* into the biscistronic pESC -URA for expression in *S. cerevisiae*. Six micrograms of yeast extracts were assayed in a plasmid DNA relaxation assay for 30 min at 37°C (as described under “Experimental Procedures”). Reaction products were resolved in agarose gel and subsequently visualized by ethidium bromide staining. The relative position of the negatively supercoiled DNA substrate is indicated by Sc, whereas the ladder of relaxed DNA topoisomer bands is labeled R. B) Scheme of the *LdTopIL* and *LdTopIS* deletions made on the non conserved extensions of each subunit and the combinations made between them. Deletions were named according to the amino acid position where the protein was truncated. Distances represented in the drawings are not in scale. C) 10% SDS-PAGE showing the purity of the different truncations used in the study.

The different constructs were obtained by cloning the distinct PCR fragments in the bis-cistronic vector pESC -URA using current molecular biology techniques. The restriction sites for *LdTopIL* gene were *BamH I* and *Xho I*, whereas *Not I* and *Spe I* were chosen for *LdTopIS* gene. Figure 1 (A and B) shows the lineal structure of the bi-subunit LdTopIL/S, describing the different truncations performed on LdTopIL and LdTopIS subunits (scheme is not at scale).

The sequence of the primers used for gene amplification and their positions are listed in Table 1. The PCR reaction contained 20 ng of plasmid *pSK-LdTopIL* or *pSK-LdTopIS* as template, 250 ng of each oligonucleotide, 100 µM dNTPs, 5 µl of 10×*Pfu* buffer and 2.5 units of *Pfu*-polymerase for a total volume of 50 µl. The cycle was composed by a 5 min step at 94°C, followed by 29 cycles at 94°C for 1 min, 66°C for 1 min and 72°C for 1 min and ending with 10 min at 72°C. The genes were subcloned into the *BamHI/XhoI* sites for the *LdTopIL* subunit and *NotI/SpeI* for *LdTopIS* in the yeast expression vector pESC -URA under control of promoters GAL1 and GAL10 respectively.

**Table 1:** Sequences of the primers used in this study to truncate LdTopIB.

26–635 Forward	CGGGATCC ATG GAG GAC CTG AAC TGG TGG
26–635 Reverse	CCGCTCGAG CTA CAC CCT CAA AGC TGC
1–468 Forward	CGGGATCC ATG AAG GTG GAG AAT AGC
1–468 Reverse	CCGCTCGAG TCA CTG CAT CAT CTG CAA CTTG
26–490 Forward	CGGGATCC ATG GAG GAC CTG AAC TGG TGG
26–490 Reverse	CCGCTCGAG CTA CTT CTT GGC GGT CAC CTC CGC CTT
26–519 Forward	CGGGATCC ATG GAG GAC CTG AAC TGG TGG
26–519 Reverse	CCGCTCGAG CTA CTC CTC CGT GCC GTA GCT CTC CAG
26–549 Forward	CGGGATCC ATG GAG GAC CTG AAC TGG TGG
26–549 Reverse	CCGCTCGAG TCA GGC GCC CGA CGT GGA TTTC
26–561 Forward	CGGGATCC ATG GAG GAC CTG AAC TGG TGG
26–561 Reverse	CCGCTCGAG CTA GGC GGC CCT CTT CTT GCC AG
26–575 Forward	CGGGATCC ATG GAG GAC CTG AAC TGG TGG
26–575 Reverse	CCGCTCGAG CTA GCT CAA CAC CTT TCC ACC CTT C
169–262 Forward	ATAAGAATGCGGCCGC ATG CCC ACG CTG GTG CCT CCG CGT CCT
169–262 Reverse	GACTAGT GGA GAT CAA GTC GCG C
200–262 Forward	ATAAGAATGCGGCCGC ATG GAG GAG AAC ATC ATT CGC ATC
200–262 Reverse	GACTAGT GGA GAT CAA GTC GCG C
210–262 Forward	ATAAGAATGCGGCCGC ATG AAC AGG CTG TGT CG
210–262 Reverse	GACTAGT GGA GAT CAA GTC GCG C

### Yeast expression system

**Figure 2:**
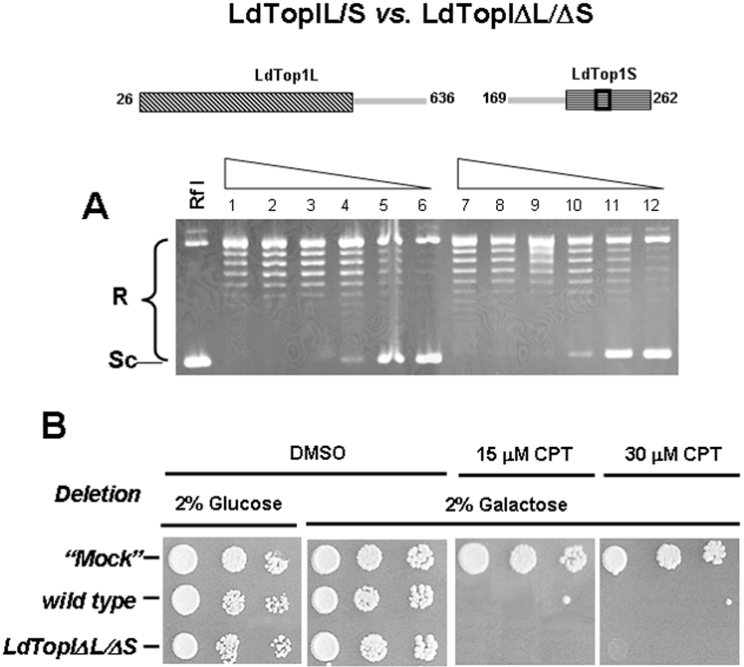
Topoisomerase activity and CPT sensitivity of the double truncation LdTopIΔL/ΔS purified from yeast extracts. A) Protein-dilution relaxation assay of LdTopIΔL/ΔS truncation (lanes 7 to 12) compared to wild type (lanes 1 to 6). 0.2 Micrograms of purified protein and a two-fold dilution series were incubated with 0.3 µg of RfI Φ X174 supercoiled DNA for 30 min at 37°C. B) Spot tests showing the sensibility to CPT of MBY3 yeast strain transformed with the “empty” pESC-URA vector (“mock”) or carrying the wild type or the double deletion *LdTopIΔL/ΔS* Exponentially growing cells in glucose, previously transformed with vectors carrying the corresponding topoisomerase encoding genes, were serially 10-fold diluted starting from OD_595_ = 0.3. Five microliters were spotted on selective media under repressed (2% glucose) or induced (2% galactose) conditions, in the presence or absence of 15 µM and 30 µM CPT. “Mock” assays were performed under similar assay conditions using the pESC -URA vector without any insert C) Dose-response curves of these transformed yeast to different concentrations of CPT. A, B and C are pictures representatives of multiple experiments. D is the average of four independent experiments.

*S. cerevisiae* strains EKY3 and MBY3 were transformed with the constructs showed in Figures 1 and 2, using the lithium acetate method [19]. The promoted GAL expression vectors carrying the URA3 selection marker were maintained by selection in synthetic complete S.C. uracil- media. At least four independent clones were selected from each transformation. Prior to the 6-h induction with 2% galactose, each starter culture was incubated in SC ura- 2% raffinose medium to eliminate any traces of glucose that might inhibit the GAL promoter expression. The cells were harvested by centrifugation, washed and resuspended in TEEG buffer (50 mM Tris-HCl pH 7.4, 1 mM EDTA, 1 mM EGTA, 10% glycerol) supplemented with 0.2 M KCl and protease inhibitors cocktail [0.1 mg/ml sodium fluoride, 0.8 mg/ml sodium bisulphite, 2×Complete Mini® (Roche Molecular Biochemicals)]. Cell extracts were prepared by disruption with acid-washed glass beads according to a previously described procedure [20]. Briefly, cells were subjected to one freeze/thaw cycle at −80°C, lysed by vortexing with 425–600 µm glass beads and the extracts were cleared by centrifugation at 15000×g for 30 min at 4°C.

### Protein purification

The yeast cultures expressing different deletions were harvested, washed and disrupted as above. The cell extracts were subjected to double-ammonium sulfate fractionation (35 and 75% saturation) and the supernatant from the second precipitation was loaded onto a phosphocellulose (P-11) column. The recombinant proteins (wild-type and truncations) were eluted at 4°C with a discontinuous gradient of KCl (0.2, 0.4, 0.6, 0.8 and 1 M) in TEEG buffer, supplemented with 0.1 mg/ml sodium bisulphite, 0.8 mg/ml NaF and 0.2× protease inhibitors cocktail. The active fractions were loaded onto a phenyl-sepharose column and eluted with a discontinuous inverse gradient of KCl (1, 0.8, 0.6, 0.4 and 0.2 M). In order to reach an appropriate concentration for the different *in vitro* assays, the eluate from the phenyl-sepharose column was concentrated by Microcon YM-30 (Millipore®). To store it, 40% glycerol was added to preserve the activity and to keep in a −20°C freezer. Protein was determined by the Bradford method [21].

### DNA relaxation assays

DNA topoisomerase I activity was assayed by the relaxation of negatively supercoiled plasmid DNA. The reaction mixture in a total volume of 20-µl contained 0.3 µg of supercoiled Rf I ΦX 174 DNA, 10 mM Tris-HCl buffer pH 7.5, 5 mM MgCl_2_, 0.1 mM EDTA, 15 µg/ml bovine serum albumin, 50 mM KCl and various concentrations of truncated specimens or wild type proteins. The reaction mixtures were incubated for 30 min at 37°C and stopped adding up to 1% SDS. The enzyme was digested with the addition of 2 µg of proteinase K, incubating for 1 h. The extent of plasmid DNA relaxation was assessed by electrophoresis in a 1% agarose gel in 0.1 M Tris borate EDTA buffer pH 8.0 at 2 V/cm for 14 h. The gels were visualized under UV illumination after being stained with ethidium bromide (0.5 mg/ml) and a posterior electrophoresis in the presence of 0.1 mg/ml ethidium bromide, in order to separate the relaxed topoisomers from the nicked forms [22].

## Results

### *Leishmania donovani* DNA-topoisomerase IB (LdTopIL/S)

According to previous reports, LdTopIL/S was cloned, functionally expressed and purified using a yeast heterologous system [4]. The linear schematic representation of Fig 1A shows that LdTopIL contains a non-conserved N-terminal extension (startMet-Glu43), followed by a region which resembles the hTopI “core” domain (Arg44-Ser456). Beyond Ser456 the homology with hTopI is lost dramatically and a non conserved C-terminal extension (Val457-Val635) displays no apparent function in topoisomerase activity. This region however, contains a long tail enriched in lysine residues, which may be putative nuclear localization signals (NLS).

The small leishmanial subunit LdTopIS contains a large non-conserved N-terminal extension (startMet-Lys211) enriched in serine residues which precedes to a C-terminal end closely homologous to the highly conserved C-terminal TopIB domains in other eukaryotes.

### Combinatorial expression of LdTopIL, LdTopIS deleted genes in a topoisomerase deficient yeast strain

In order to know the role played by the non-conserved extensions of both LdTopIL and LdTopIS in topoisomerization of supercoiled DNA, a gradual deletion approach was carried out. For this purpose gradually deleted *LdTopIL* genes were cloned into the pESC -URA multiple cloning site driven by GAL1 promoter, remaining the unchanged *LdTopIS* gene into the multiple cloning site driven by GAL10 promoter (Fig 1A). The truncated proteins encoded by these genes lack the 26 first amino acids from the N-terminal domain (LdTopIΔL from now on) and three increasing length segments from the C-terminal end called LdTopIΔ^467^L, LdTopIΔ^490^L and LdTopIΔ^519^L. Relaxation of supercoiled DNA using yeast extracts that express the above-mentioned genes showed that, with exception of LdTopIΔL, which conserved full enzymatic activity, the other three deletions expressed non active topoisomerase specimens and thereby the removed peptides were considered essential to unwind DNA or to keep both subunits together in the heterodimer.

A similar approach was carried out deleting peptide segments from the N-extension end of LdTopIS. In a very recent report, the authors showed those truncations missing the entire N-terminal extension (called truncation E210end) or containing a short coil portion of this region (called truncation E200end) lacked any identifiable topoisomerase characteristics, in terms of DNA relaxation or CPT sensitivity when co-expressed with the large subunit [23]. A shorter 169 amino acids truncation (LdTopIΔS from now on) was then designed. LdTopIΔS conserves the α-helix comprised between amino acids 190 to 208 intact, and an extra sequence which has no identifiable spatial arrangement (amino acids 169 to 189). This truncated protein relaxed supercoiled DNA at similar extent that wild type and was inhibited by CPT (data not shown).

### Selection of functional topoisomerase truncations

Once regions involved in DNA relaxation were delimited within each enzymatic subunit, we proceeded to purify active truncated proteins with no distinguishable topoisomerase activity from wild type using large scale yeast cultures as described in Materials and Methods (Fig 1 B, C).

In a first approach we determined the activity of single truncations at N-terminal end of each subunit. 0.2 µg of each LdTopIΔL/S and LdTopIL/ΔS truncations and two-fold serially dilutions were assayed using 0.3 µg of supercoiled close circular DNA from the virus ΦX-174 (Rf I) DNA as substrate. No differences were found between these enzymatic specimens and wild-type in terms of topoisomerase activity (data not shown).

The combinatory expression of both deleted genes *LdTopΔL/ΔS*, served to generate a double truncated protein from EKY3 yeast deficient strain. After induction with galactose and purification according to the methods previously described, a relaxation assay was performed under the standard assay conditions, using similar protein concentrations of the purified wild type topoisomerase as relaxation control. Fig. 2A shows no differences in relaxation activities and distributive pattern between this double truncation and the wild type. For these reasons this protein specimen was used as minimal structure for further deletion studies into the large subunit.

We have analyzed the effect of CPT on a yeast-deficient topoisomerase I strain (MBY-3: TopI Δ::TRP1 rad52 Δ::LEU2) transfected with the bidirectional pESC -URA plasmid bearing the combination of double deleted *LdTopIL* and *LdTopIS* genes. Spot tests depicted in Fig. 2B show that after 2% galactose induction, CPT was as lethal for the cultures transformed with deletions *LdTopIΔL/ΔS* as for the wild type. Yeasts transformed with the “empty” vector (“mock”) were used as control, showing no response to CPT. These results agree well with the relaxation assays, showing that topoisomerase specimens expressed by yeasts were functional *in vivo*.

### Searching for a minimal structure in LdTopIL

Once the double LdTopIΔL/ΔS truncation showed no differences in relaxation activity with respect to the wild type phenotype, a further gradual deletion study was carried out in the C-extension end of LdTopIL. It had been established from the results of Fig. 1 that yeasts transformed with deletions containing up to 52 amino acids upwards the end of the putative “core” domain, were not able to relax supercoiled DNA in a standard DNA-relaxation assay. It seems obvious that the amino acids comprised in this region (Gln-467 to Glu-519) should be relevant for enzyme integrity and thereby the truncations performed in this second study might contain them. The new topoisomerase specimens prepared within the region comprised between Ala548 to the protein end were: LdTopIΔ^548^ΔL, LdTopIΔ^561^ΔL and LdTopIΔ^575^ΔL, respectively and served to narrow the region involved, in any extent, in topoisomerization or protein-protein binding. These constructs were cloned together with *LdTopIΔS* deletion in the biscistronic pESC -URA vector, expressed and purified from EKY3 transformed yeast according to the above-mentioned method.

**Figure 3:**
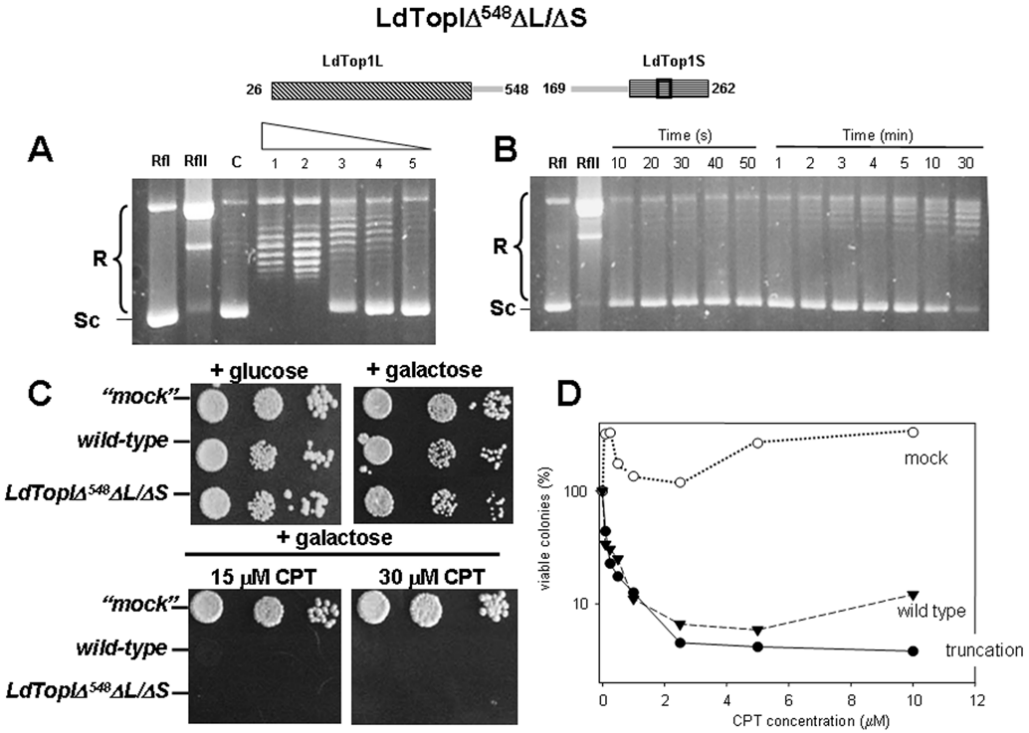
Topoisomerase activity of purified truncation LdTopIΔ^548^ΔL/ΔS. A) Two-fold serial dilutions of the triple truncation LdTopIΔ^548^ΔL/ΔS were assayed in a plasmid DNA relaxation assay for 30 min at 37°C. B) Time-course experiment of topoisomerase activity using 0.2 µg of truncation LdTopIΔ^548^ΔL/ΔS per reaction 0.2 Micrograms of purified protein and a two-fold dilution series were incubated with 0.3 µg of RfI Φ X174 supercoiled DNA for 30 min at 37°C. Relaxation rate was compared with lane RfII which includes 0.3 µg of Φ X174 relaxed DNA C) Spot tests showing the sensibility to CPT of MBY3 yeast strain transformed with the “empty” pESC-URA vector (“mock”) or carrying the wild type LdTopIL/S genotype and deletion LdTopIΔ^548^ΔL/ΔS D) Dose-response curves of these transformed yeast to different concentrations of CPT. A, B and C are pictures representatives of multiple experiments. D is the average of four independent experiments.

LdTopIΔ^548^ΔL/ΔS conserves a 18 amino acids length α-helix (Glu520 to Val538) which resembles the secondary structure of yeast topoisomerase I and a putative NLS (Arg476 to Lys491). Figure 3A shows the activity of the truncation LdTopIΔ^548^ΔL/ΔS in a DNA relaxation assay using similar conditions to those employed in Fig. 2. It is remarkable a weak but clear relaxation activity, as well as a slow appearing of DNA topoisomers detected in time-course experiments (Fig. 3B). Spot test experiments show however, no differences between LdTopIΔ^548^ΔL/ΔS and wild type in CPT sensitivity (Fig 3C), data which were reinforced in dose-response experiments (Fig 3D). Altogether it can be concluded that despite LdTopIΔ^548^ΔL/ΔS is a very weak active protein in relaxing supercoiled DNA, it is a DNA-cleaving enzyme and thereby susceptible to be poisoned by CPT.

**Figure 4:**
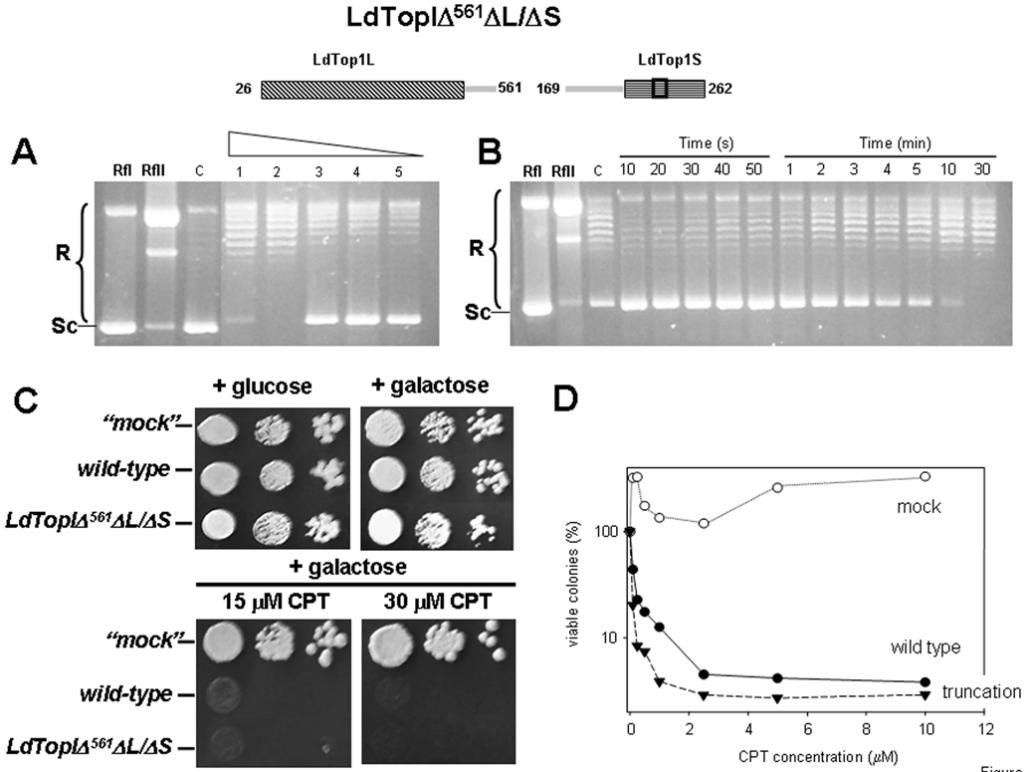
Functional expression of purified LdTopIΔ^561^ΔL/ΔS truncation. A) Two-fold serial dilutions of truncation LdTopIΔ^561^ΔL/ΔS were assayed in a plasmid DNA relaxation assay for 30 min at 37°C. B) Time-course experiment of topoisomerase activity using 0.2 µg of truncation LdTopIΔ^561^ΔL/ΔS per reaction C) Spot tests showing the sensibility to CPT of MBY3 yeast strain transformed with the “empty” pESC-URA vector (“mock”) or carrying the wild type LdTopIL/S genotype and deletion LdTopIΔ^561^ΔL/ΔS D) Dose-response curves of these transformed yeast to different concentrations of CPT. A, B and C are pictures representatives of multiple experiments. D is the average of four independent experiments. Relaxation rate was compared with lane RfII which includes 0.3 µg of Φ X174 relaxed DNA. The “c” lane corresponds to an activity control, using a commercial topoisomerase.

**Figure 5:**
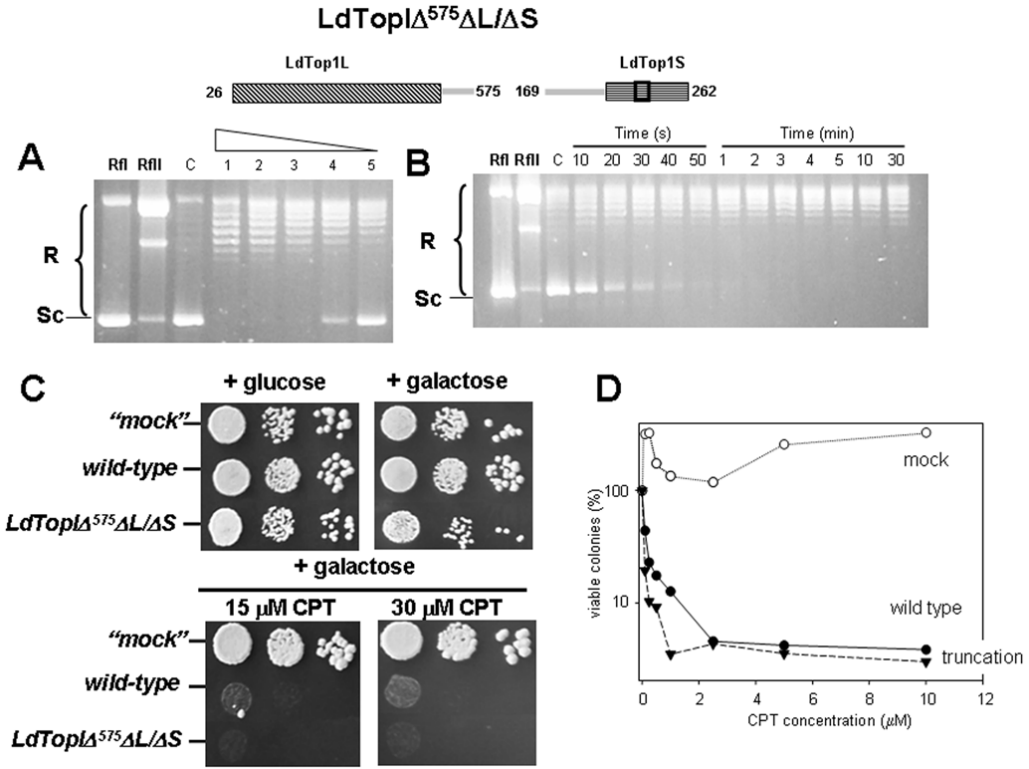
Enzymatic topoisomerase activity of LdTopIΔ^575^ΔL/ΔS truncation. A) Protein dilution relaxation assay: Two-fold serial dilutions of truncation LdTopIΔ^575^ΔL/ΔS were assayed in a plasmid DNA relaxation assay for 30 min at 37°C. B) Time-course experiment of topoisomerase activity using 0.2 µg of truncation LdTopIΔ^575^ΔL/ΔS per reaction C) Spot tests showing the sensibility to CPT of MBY3 yeast strain transformed with the “empty” pESC-URA vector (“mock”) or carrying the wild type LdTopIL/S genotype and deletion LdTopIΔ^575^ΔL/ΔS D) Dose-response curves of these transformed yeast to different concentrations of CPT. A, B and C are pictures representatives of multiple experiments. D is the average of four independent experiments. Relaxation rate was compared with lane RfII which includes 0.3 µg of Φ X174 relaxed DNA. The “c” lane corresponds to an activity control, using a commercial topoisomerase.

LdTopIΔ^561^ΔL/ΔS is an intermediary 14 amino acids longer which displays a putative NLS. Time-course (Fig. 4B) and protein-dilution assays (Fig. 4A) carried out with purified truncated protein show slow but detectable topoisomerase activity. Spot test (Fig 4C) and dose-response (Fig 4D) experiments show no differences in CPT sensitivity respect wild type, suggesting that DNA-cleaving activity of this truncation is very likely. Finally LdTopIΔ^575^ΔL/ΔS (Fig. 5) was performed in order to assign a role to two lysine-enriched regions (Lys549 to Leu565 and Lys576 to Glu592) mostly arranged in α-helix and with putative NLS motifs (according to PSORT-II on-line software). Protein dilution (Fig. 5A) and time-course (Fig. 5B) assays show no differences in relaxation activity between them and a similar CPT sensitivity than the wild type (Fig. 5C and 5D), suggesting that these regions are disposables for activity although it may be relevant to drive the enzyme to the nucleus compartment.

## Discussion

The existence of an unusual heterodimeric type IB topoisomerase in trypanosomatids was pointed out by our group in 2003 using a genomic approach in *L. donovani*. Since then [4] several reports have confirmed the presence of multimeric topoisomerases in other trypanosomatids like *Trypanosoma brucei* [5] and *T. cruzi* (*Trypanosoma cruzi* Genome Project http://www.tigr.org/tdb/e2k1/tca1/). This remarkable finding confirmed the results obtained in a previous report describing a putative topoisomerase lacking the C-terminal domain in *L. donovani* [24] and indicated that the small subunit had been ignored in early studies [25]. Once resolved the primary amino acidic sequence of both subunits, it is of a paramount importance to know how these monomers are assembled to build up a functional heterodimer displaying fully relaxation activity and susceptible to be inhibited by CPT. It is of interest therefore, to identify structural insights indispensable for protein-protein interaction between large and small subunits.

The alignment of the amino acid sequence of LdTopIL/S with the human counterpart as well as its crystalline configuration show that overall, structure and catalytic machinery of the human and leishmanial enzymes are conserved, despite the fact that one of them is a monomer and the other is a heterodimer [26]. From this comparison it is very likely that the N-terminal and core domains are placed at the LdTopIL subunit and the C-terminal end within the small LdTopIS subunit. However, with the data managed at present, it is not easy to assign a putative linker domain to a particular region of the bi-subunit leishmanial enzyme. The importance of this region is not only due to be a simple connector between core and C-terminal domains [27], but it contributes to DNA binding and CPT inhibition, probably by slowing down the religation step of the nicking-closing reaction [28]-[29].

The evidences presented here indicate that long extensions placed at C-terminal end of LdTopIL and at N-terminal end of LdTopIS, respectively are dispensable in terms of relaxation activity and sensitivity to CPT. Moreover, the combinatorial association of some of these truncations restored a fully active enzyme which retained unaltered the cleavage and religation functions associated to any topoisomerase, in similar terms of wild-type. Results obtained in the present work show that at least a 94 amino acids long C-extension comprised between Gln467 to Ala548, are required to retain measurable relaxation activity and CPT sensitivity. Despite this extension lacks of any amino acid clearly involved in relaxation activity, it conserves a high pI which would permits the ionic interaction with the small subunit to hold the subunits together [3] and a putative NLS, as well. Further sequential deletions of the amino acids placed between truncations LdTopIΔ^561^ΔL/ΔS and LdTopIΔ^575^ΔL/ΔS, sited at the C-terminal end of the large subunit, showed that they were unnecessary for topoisomerization, but they contained multiple putative NLSs required to drive the assembled protein to the nucleus. Due to these NLS signals are placed at the C-terminal extension of the LdTopIL subunit only, it is thereby very likely that the enzyme assembly takes place in the cytosol before translocation to the nuclear compartment [3].

With regards to the small subunit, the N-extension domain comprising the first 169 amino acids seems to be unnecessary for relaxation and CPT sensitivity. However a further truncation up to Asn210 led to a neglect of interaction between subunits and topoisomerase activity. From the results obtained in this paper we can conclude that this 169 N-terminal extension of LdTopIS is not required either for the interaction between monomers or relaxation and DNA cleavage activities. A recent report shows the existence of a RPPVVSR motif within the small subunit (amino acids 175 to 182) required for relaxation activity and CPT sensitivity, thereby a minimal putative linker should contains these amino acids to fulfill topoisomerase activity [23].

Strikingly the minimal combined truncation reconstituted LdTopIΔ^548^ΔL/ΔS fully conserved the sensitivity to CPT. According to these results it may stated that the sensitivity to this class I inhibitor resides into amino acids of the core and C-terminal domains as well as in the RPPVV motif contained into the N-terminal extension of the small subunit [23]. Marquis and co-workers have shown that the resistance to CPT displayed by *L. donovani* LdRCPT.160 strain is mediated by two amino acid substitutions within the core domain of the large subunit (Gly185Arg and Asp325Glu) resulting from two single nucleotide mutations. In addition, authors observed a decrease in DNA relaxation, presumably due to the presence of these mutations [30].

Once the dispensable regions are delimited in both subunits, an essential question arises: what is the role of the long serine-enriched region placed at LdTopIS? It has been proposed that this region is suitable to phosphorylation and may participate in the proteolytic breakdown of the enzyme after CPT exposure [31]. Post-translational down-regulation of the human enzyme occurs with the hyperphosphorylated enzyme and it is replication independent [32]. Topoisomerase degradation by 26S proteasome may increase tolerance to DNA cleaving poisons [33] and facilitate the DNA repairing activity of tyrosyl phosphodiesterase [34].

Altogether this study gives structural insights of the role played by the C-extension end of the LdTopIL in its protein-to-protein interaction with the small LdTopIS to build up a fully functional enzyme. The heterodimeric nature of topoisomerase IB in trypanosomatids provides for functional complementation using individual truncated fragments. It is remarkable the lack of function played by at least 169 amino acids located within the N-terminal extension of LdTopIS, as well as those placed beyond Ala-548 of the large LdTopIL. Further truncation from the large subunit results in a stepwise loss of activity up to Glu-519, truncation deficient in relaxation activity at all. A very recent finding from another lab concluded that the amino acids 39–456 of large subunit and 210–262 of small subunit constitute the minimal interactive portion of the bi-subunit leishmanial enzyme conserving relaxation activity [35]. However the authors fail in describing a true putative linker since the enzymatic activity of this enzymatic specimen is almost undetectable as well as its sensitivity to CPT.

This paper provides structural insights into the N- and C-extensions involved in the interaction between protomers to build up a fully active topoisomerase in Leishmania parasites. These elements are not only required to reconstitute a putative linker domain, but also contain unexplored nuclear driving signals and potential post-translational motifs for down-regulation. Only a deeper knowledge of the structural design of these non-conserved regions will help to a full understanding of topoisomerase mechanisms in these ancient eukaryotes.
